# Hospital Responsibilities—Direct and Indirect

**Published:** 1916-04-08

**Authors:** 


### HOSPITAL RESPONSIBILITIES—DIRECT AND INDIRECT.

The main and primary duty of every hospital is, of course, the efficient treatment of the individual patients. If there is failure here, no other achievement may spell compensation. Hence the visiting staff have a plain responsibility, which they themselves would be the last to question. At the same time, out of the opportunities of hospital work arise other responsibilities, and by these in some measure each institution comes to be judged. The principal of these on the professional side is the record of clinical and pathological facts, and of the doctrinal and practical lessons which arise out of such facts. The wards and laboratories of a hospital form a field of natural history where the phenomena of disturbances known as diseases are exhibited, and in which these phenomena can be observed and studied. The members of the staff have free access to such collections, and from this it follows that upon them falls the duty of utilising their positions to the best advantage. Admittedly their first duty is the care of the patients. But beyond this comes the question of additions to knowledge, and only when the response to this is satisfactory can it be said that the hospital stands beyond reproach. No doubt it is very difficult to erect a standard by which all may be judged, but beyond question an institution which shows in this respect a barren record comes under condemnation. What is true of the institution is true especially of the staff, and indeed in the last analysis the one is identical with the other. So far has this general statement been recognised that at times there have been suggestions that at stated intervals the work and position of each member of the staff should be brought under review for the purpose of determining whether or not his appointment should be continued. The proposal has never taken practical shape because no one has ever been able to devise a valid standard or to organise a tribunal whose verdict would be recognised as both competent and just. All the same it remains true that members of hospital staffs enjoy great opportunities and may be reasonably asked to show what they are doing with them. That as a whole they fall below the level of other varieties of professional workers we do not for a moment believe, but this does not mean that there is not room for improvement in many individual instances.

As matters stand at present we have to be content with the virtue of the average, and we admit that we see no means of escape from this position. Unless in the case of some very gross and obvious neglect, lay governors cannot pretend to judge professional responsibilities, and members of a staff could never be persuaded to make formal reports on one another. Nor would a judgment from an outside authority be readily welcomed, even if any such authority could be secured. Thus the matter must in the end be left with the individual, though indirectly it may be tempered by the pressure of an educated professional opinion. If ever our hospitals cease to be independent units, and become organised into a system, efficiency would then lead to promotion, and neglect to supersession. But such a scheme is not within the limits of practical politics, and we are far from suggesting that it has not disadvantages of its own. In the meantime, it is well to keep up the general expectation that those who hold special positions should justify their opportunities.

